# Highly Transparent Phase Change Smart Windows Enabled by Refractive-Index-Matched n-Octadecane@SiO_2_ Microcapsule Composites

**DOI:** 10.3390/nano16110648

**Published:** 2026-05-22

**Authors:** Fusen Yang, Zhixing Zhang, Yiyu Feng, Mengmeng Qin, Wei Feng

**Affiliations:** 1 Tianjin Key Laboratory of Composite and Functional Materials, School of Materials Science and Engineering, Tianjin University, Tianjin 300350, China; fsyang@tju.edu.cn (F.Y.); zhangzx92@tju.edu.cn (Z.Z.); fengyiyu@tju.edu.cn (Y.F.); 2 State Key Laboratory of Precious Metal Functional Materials, Tianjin University, Tianjin 300350, China

**Keywords:** microcapsule, phase change, thermal management, transparency

## Abstract

The development of phase change materials (PCMs) for window applications with both high optical transparency and effective temperature regulation is crucial for passive energy saving. However, liquid leakage during phase transition and enhanced interfacial light scattering often cause fluctuations in optical transmittance and deterioration of image clarity. To address these challenges, a highly transparent phase change composite was constructed via a microencapsulation strategy. Submicron core–shell microcapsules were fabricated using n-octadecane as the core and silica as the shell, enabling effective encapsulation of the liquid PCM component. The resulting microcapsules exhibited a high melting enthalpy of 155.3 J g^−1^. They were subsequently homogeneously dispersed within a refractive-index-matched polymer matrix, mitigating light scattering during phase transition by reducing interfacial refractive index mismatch. The composite exhibited favorable thermal energy storage capability and transmittance performance, with a visible light transmittance of 83.75% and a transmittance fluctuation of only ~5% before and after phase transition. After 100 thermal cycles, the optical attenuation remained as low as 0.35%, demonstrating excellent cycling stability. This work provides a new strategy for balancing optical transparency and phase change function, with potential applications in smart windows and flexible electronics.

## 1. Introduction

In the 21st century, building energy consumption accounts for approximately 30–40% of global energy use, with heating, ventilation, and air conditioning systems consuming nearly half of a building’s total energy [1,2,3]. As the most typical transparent interface in building envelopes, windows serve both lighting and visual needs, while also being the most active channel for solar radiation and heat exchange between the interior and exterior. However, their limited solar radiation control capability often leads to significant heat loss [4,5]. Therefore, developing smart window materials that combine high optical transparency with effective thermal management is crucial for reducing building energy consumption and enhancing indoor thermal comfort.

Phase change materials (PCMs) can undergo reversible changes in crystal structure, providing a passive temperature regulation path for building thermal management without additional energy consumption [6,7]. Depending on their composition and phase transition mechanism, PCMs include inorganic types characterized by optical constant changes and organic types based on latent heat storage. The former can induce significant changes in refractive index and extinction coefficient in the solid state and has been applied in fields like optical storage and filtration [8,9,10]. However, they are often costly, with limited processing and resource attributes, and may pose environmental burdens [11,12]. More importantly, many inorganic phase change systems exhibit inherent absorption in the visible light spectrum, making it difficult to meet the high transparency requirements for building windows [13,14]. In contrast, organic PCMs such as paraffin and fatty alcohols can be designed with molecular structures that place the phase transition temperature near room temperature or thermal comfort zones while exhibiting minimal absorption in the visible light range [15,16,17]. They offer advantages such as low cost, good biocompatibility, and environmental friendliness, making them more promising candidates for transparent thermal management window materials [18,19]. However, organic PCMs are prone to leakage during the solid–liquid phase transition [20]. Additionally, differences in refractive indices between the solid and liquid phases and the microstructural evolution during phase changes often lead to enhanced light scattering, causing optical instability such as transmittance fluctuations, thus limiting their further application in “transparent and sustainable” window scenarios [21,22].

To address the challenges of leakage and optical stability in organic PCMs, researchers have proposed various material design strategies. Microencapsulation is a representative method: Guo et al. prepared SiO_2_@n-octadecane phase change nanocapsules with controllable size and structure, achieving high phase change enthalpy and good thermal stability [23]; Ju et al. developed n-octadecane/SiO_2_ nanocapsules via a sol–gel method and incorporated them into cement-based materials, demonstrating high latent heat, good cycling durability, and enhanced thermal storage performance [24]. Zhang et al. modified the SiO_2_ shell with silane coupling agents to improve interfacial bonding and anti-leakage properties while enhancing the microcapsules’ enthalpy retention capability [25]. Zhang et al. further integrated room-temperature adaptive silica-shell/oil-core phase change microcapsules with cellulose fibers to construct a micro/nanostructured composite film, achieving adaptive thermal regulation and efficient sustainable cooling [26]. However, the transparency of phase change microcapsules has not been thoroughly studied yet. A transparent polymer matrix can be used to regulate the transparency of composite materials. Vallan, Lorenzo et al. embedded paraffin particles in a PVA coating to exploit refractive index mismatch induced by phase transition to achieve thermochromic shading effects [27]; Montanari, Céline et al. introduced PEG-based PCMs into transparent wood templates and achieved relatively stable and higher transmittance before and after the phase transition [28]. In recent years, ultraviolet curing processes have been introduced into the preparation of phase change composite materials. Lee et al. introduced epoxy-functionalized acrylate monomers into octadecane acrylate or octadecane methacrylate systems, using UV curing to prepare laminated glass and films, enhancing glass adhesion and impact resistance while achieving thermal storage functionality [29]. Overall, while progress has been made in suppressing leakage and enhancing mechanical properties in existing PCM-based smart windows, most systems still exhibit significant scattering enhancement and transmittance fluctuations at high temperatures or across phase transition ranges, thereby weakening indoor visual continuity and affecting practical user experience.

In this study, a transparent phase change composite material was designed and prepared by microencapsulation and UV curing processes. Specifically, core–shell structured n-octadecane@SiO_2_ microcapsules were prepared and evenly dispersed into acrylate monomers. UV-induced crosslinking forms a transparent polymer network. The SiO_2_ shell was innovatively used to physically encapsulate the PCMs to suppress leakage while reducing phase transition-induced light scattering through refractive index matching and interfacial compatibility design between the shell and polymer matrix. By adjusting the composition, microstructure, particle size, and composite process parameters of microcapsules, composite materials with both transparency and phase change functions were developed. The final phase change composite not only maintains a visible light transmittance of up to 83.75% at 550 nm but also exhibits a transmittance change of only 5% before and after phase transition, with light transmittance degradation being as low as 0.35% after 100 thermal cycles. This transparent phase change composite material has potential application value in future fields such as smart windows and flexible electronics.

## 2. Materials and Methods

### 2.1. Materials

n-Octadecane, hexadecyl trimethyl ammonium bromide (CTAB) and ammonia solution (NH_3_·H_2_O) were purchased from Shanghai Mirac Chemical Technology Co., Ltd. (Shanghai, China). Tetraethyl orthosilicate (TEOS) and vinyltriethoxysilane (VTES) were purchased from Shanghai Meck Biochemical Technology Co., Ltd. (Shanghai, China). Dodecyl acrylate, n-octyl acrylate, n-butyl acrylate, and 2,2-diethoxyacetophenone (DEAP) were purchased from TCI (Shanghai) Development Co., Ltd. (Shanghai, China). Ethylene glycol dimethacrylate (EGDMA) was purchased from Shanghai Aladdin Biochemical Technology Co., Ltd. (Shanghai, China).

All reagents were of analytical grade and used as received without further purification.

### 2.2. Preparation of n-Octadecane@SiO_2_ Microcapsules

**Figure 1:**
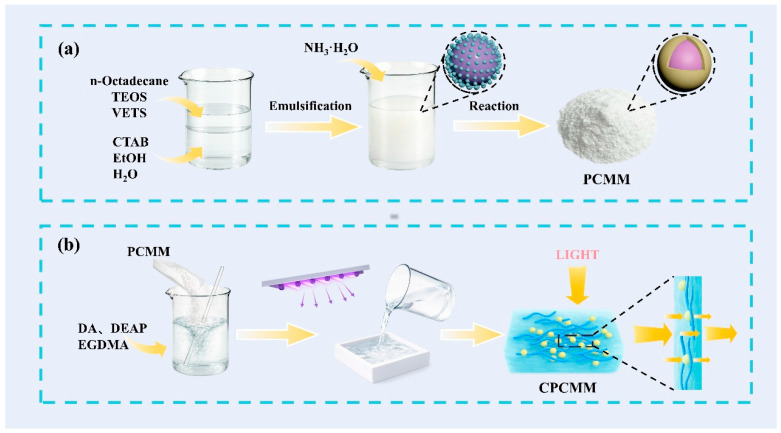
Schematic illustration of the preparation process for (**a**) PCMM and (**b**) CPCMM.

The preparation process of n-octadecane@SiO_2_ microcapsules is shown in Figure 1 and is achieved through the interfacial hydrolysis and condensation reaction of TEOS in a microemulsion system. First, n-octadecane and TEOS were mixed in a beaker, and then CTAB, VTES, deionized water, and ethanol were sequentially added. The mixture was heated to 60 °C until all solids were dissolved, forming a homogeneous solution. The resulting solution was then subjected to high-speed homogenization at 35 °C and 10,000 rpm for 3 min, followed by ultrasonic treatment at the same temperature for 10 min to obtain a stable microemulsion system. The microemulsion was then transferred to a three-neck flask equipped with a mechanical stirrer and a thermostatic oil bath. The mixture was stirred at 60 °C and 600 rpm while NH_3_·H_2_O was slowly added to initiate the hydrolysis and condensation of TEOS at the oil–water interface. After 6 h of reaction, the system was centrifuged at 6000 rpm for 5 min, and the supernatant was discarded. All microcapsule samples were sequentially washed three times with ethanol and three times with deionized water to eliminate unencapsulated PCMs and avoid its influence on thermal analysis. Finally, the washed product was frozen at −20 °C for 12 h and then freeze-dried for 48 h to obtain a white powder of n-octadecane@SiO_2_ phase change microcapsules, denoted as PCMM.

### 2.3. Preparation of Phase Change Composites

Dodecyl acrylate was used as the matrix monomer and mixed with the photoinitiator DEAP, crosslinking agent EGDMA, and PCMM. The mixture was thoroughly stirred at room temperature to ensure uniform dispersion of all components. The resulting mixture was then poured into pre-prepared molds and cured under ultraviolet light for 20 min. After UV curing, the n-octadecane@SiO_2_ microcapsule/dodecyl acrylate phase change composite material was obtained, denoted as CPCMM.

### 2.4. Characterization

The morphology and microstructure of Pt-coated PCMM and CPCMM were observed by field emission scanning electron microscopy (SEM, SU8600, Hitachi, Tokyo, Japan). The particle size distribution of PCMM was analyzed using Nano Measurer 1.2 software. The crystallization behavior of the samples was investigated using an X-ray powder diffractometer (XRD, XRD-6100, Shimadzu, Kyoto, Japan) in the 2θ range of 5–80°. The chemical structures of the samples were characterized using Fourier transform infrared spectroscopy (FT-IR, Nicolet IS50, Thermo Fisher Scientific, Waltham, MA, USA) in the range of 4000–400 cm^−1^. The phase transition temperature and enthalpy of the samples were measured using differential scanning calorimetry (DSC, Q20, TA Instruments, New Castle, DE, USA) under a nitrogen atmosphere at a heating rate of 10 °C min^−1^. The thermal reliability of the samples was evaluated using heating–cooling cycle tests in the temperature range of 10–60 °C. The optical transmittance of CPCMM in the visible region (380–750 nm) was measured using ultraviolet–visible spectroscopy (UV–Vis, UV-3600 Plus, Shimadzu, Kyoto, Japan). The thermal stability of n-octadecane, PCMM, and CPCMM was analyzed through thermogravimetric analysis (TGA, STA200, Hitachi, Tokyo, Japan). The tensile properties of CPCMM were tested using universal testing (UTM, UTM2203, Shenzhen Suns Technology Stock Co., Ltd., Shenzhen, China). The refractive index of the material was measured using refractive index measurement (Abbe refractometer, WYA-2WA, Shanghai Yice Instrument Equipment Co., Ltd., Shanghai, China). Solar irradiation for photothermal experiments was simulated using a xenon lamp (CEL-S500/350, Zhongjiao Jinyuan, Beijing, China). During the phase change heat-transfer process under irradiation, the temperature evolution of the samples was recorded in real time using digital multimeter measurement (Keithley 2001, Keithley, Cleveland, OH, USA) connected to a thermocouple (SIN-R9600, Sinomeasure, Hangzhou, China), while the spatial temperature distribution was monitored using infrared thermal imaging (TiX640, Fluke, Everett, WA, USA).

## 3. Results

### 3.1. Structural and Functional Design Mechanism of CPCMM Composites

In this study, phase change microcapsules comprising n-octadecane as the core material and SiO_2_ as the inorganic shell were successfully fabricated via an interfacial sol–gel method, as illustrated in Figure 1a. The detailed reaction parameters are summarized in Table S1. During the preparation process, narrow particle size distribution and uniform-sized emulsion droplets were obtained through high-speed shear combined with ultrasonic dispersion. CTAB reduced the interfacial tension and provided electrostatic repulsion to suppress aggregation [30]. Under ammonia-catalyzed conditions, TEOS underwent hydrolysis and condensation reactions, enriching at the interface and preferentially undergoing heterogeneous nucleation, thereby forming a dense, amorphous SiO_2_ shell and achieving a high encapsulation efficiency core–shell structure [31]. The constructed SiO_2_ shell effectively constrained the leakage and volume change in the core material during the phase transition, thereby enhancing the thermal stability and leakage resistance of the microcapsules. Additionally, the introduction of VTES for co-condensation with TEOS resulted in the grafting of polymerizable vinyl functional groups on the shell surface, providing reaction sites for subsequent chemical bonding with the organic matrix.

As shown in Figure 1b, the obtained microcapsules were subsequently dispersed in a dodecyl acrylate prepolymer, and the composite was fabricated through UV-initiated free-radical polymerization in which the polyacrylate matrix was strongly coupled with the microcapsules through chemical bonding. The composite material maintained high transparency during the phase transition, primarily due to the multi-scale optical collaborative design: the high refractive index matching between the amorphous SiO_2_ shell (*n* ≈ 1.46), n-octadecane core (*n* ≈ 1.45), and the crosslinked poly (dodecyl acrylate) (*n* ≈ 1.46), which fundamentally reduced the Fresnel reflection and scattering loss at the interface. The microcapsule particle size was controlled at the submicron level, avoiding optical losses dominated by Mie scattering of PCMs. The sol–gel shell surface was smooth and dense, ensuring uniform dispersion of the microcapsules and reducing scattering centers [32,33]. Although the refractive index of n-octadecane slightly changes during the solid–liquid phase transition around 30 °C, its encapsulation in the refractive index-stable SiO_2_ shell ensures that, while maintaining a submicron scale, the overall refractive index remains consistently matched. This results in the composite material exhibiting stable and reversible optical properties before and after the phase transition. Therefore, this system achieves significant phase transition enthalpy while maintaining approximately 80% visible light transmittance over multiple cycles, effectively balancing high transparency with thermal energy storage functionality.

### 3.2. Control of Microcapsule Size

In the CTAB-stabilized oil/water (O/W) system, the water/ethanol volume ratio significantly influences the assembly state of CTAB at the oil–water interface and the reaction kinetics of TEOS by altering the dielectric constant of the continuous phase and the solvation environment, thus playing a key role in regulating the initial size of the emulsion droplets and the final growth scale of the SiO_2_ shell [34]. As the ethanol ratio decreases, the dielectric constant of the continuous phase increases, reducing the electrostatic repulsion between the CTAB head groups and causing the molecular arrangement at the interface to become more compact, which favors the formation of smaller emulsion droplets and ultimately results in microcapsules with smaller sizes [35]. This trend of decreasing particle size aligns well with the design requirements of transparent phase change composites, where minimizing the size of scattering centers is crucial to effectively suppress visible light scattering.

**Figure 2:**
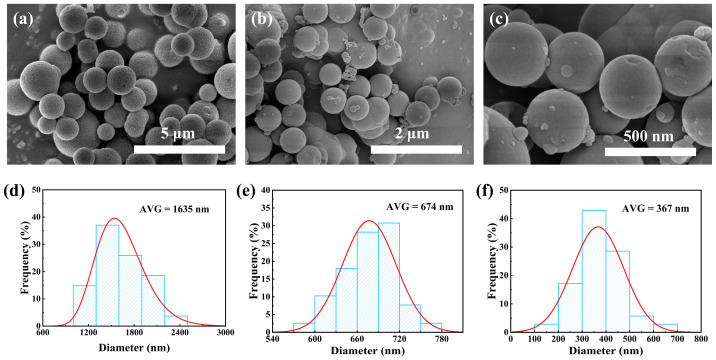
(**a**–**c**) SEM images and (**d**–**f**) corresponding particle size distributions of PCMM-1, PCMM-2 and PCMM-3.

SEM was employed to characterize the morphological evolution and particle size distribution of the microcapsules. As illustrated in Figure 2a–c, samples synthesized at water/ethanol volume ratios of 1.5:1 (PCMM-1), 2.0:1 (PCMM-2), and 2.5:1 (PCMM-3) all exhibit nearly spherical morphology with continuous and intact shells, indicating that the water/ethanol ratio can be effectively controlled within a certain range to achieve size regulation without disrupting the core–shell structure. The particle size distribution statistics (Figure 2d–f) further confirm that, as the water/ethanol ratio increases, the average particle size of the microcapsules decreases from the micron scale to the submicron scale: the average particle sizes of PCMM-1, PCMM-2, and PCMM-3 are 1635 nm, 674 nm, and 367 nm, respectively.

**Figure 3:**
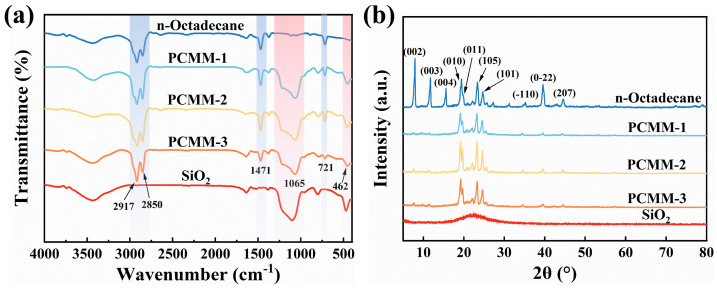
(**a**) FT-IR spectra and (**b**) XRD patterns of n-octadecane, SiO_2_, and PCMM-1–PCMM-3.

To confirm that the differences between PCMM-1, PCMM-2, and PCMM-3 were mainly due to particle size rather than changes in chemical composition or phase structure, the samples were characterized by FT-IR and XRD. As shown in Figure 3a,b, FT-IR spectra show that all microcapsule samples retain the characteristic absorption peaks of both SiO_2_ and n-octadecane: the asymmetric and symmetric stretching vibration peaks of Si-O-Si backbone at 1065 cm^−1^ and 462 cm^−1^, respectively; the –CH_2_– stretching vibration characteristic peaks of n-octadecane at 2917 cm^−1^ and 2850 cm^−1^; and the bending and wagging vibration peaks of the alkyl chain at 1471 cm^−1^ and 721 cm^−1^. The peak positions of the three samples are essentially identical, and no new absorption bands appear, indicating that the water/ethanol ratio regulation process did not introduce new chemical structures or induce significant side reactions. n-Octadecane was successfully and stably encapsulated within the SiO_2_ shell. The XRD patterns (Figure 3b) further support the structural similarity: all samples display the typical amorphous broad diffraction peaks of SiO_2_ [36]; the characteristic crystalline diffraction peaks of n-octadecane can still be clearly observed in the microcapsule samples [37], indicating that the encapsulation process preserved the crystalline characteristics of the core material. Compared to n-octadecane, the intensity of the diffraction peaks for n-octadecane in the microcapsules is slightly reduced and accompanied by broadening. This is mainly attributed to the diffraction background of the amorphous SiO_2_ shell and the slight suppression of core material crystal growth and perfection due to the confinement effect of the shell, rather than an intrinsic change in the crystalline structure [23]. The above results indicate that PCMM-1–PCMM-3 are highly consistent in chemical composition and phase structure, with only controlled differences in particle size, providing a reliable experimental basis for systematically studying the effects of microcapsule particle size on the optical transparency and thermal properties of the composites.

### 3.3. Phase-Change Behavior of the Microcapsules

**Figure 4:**
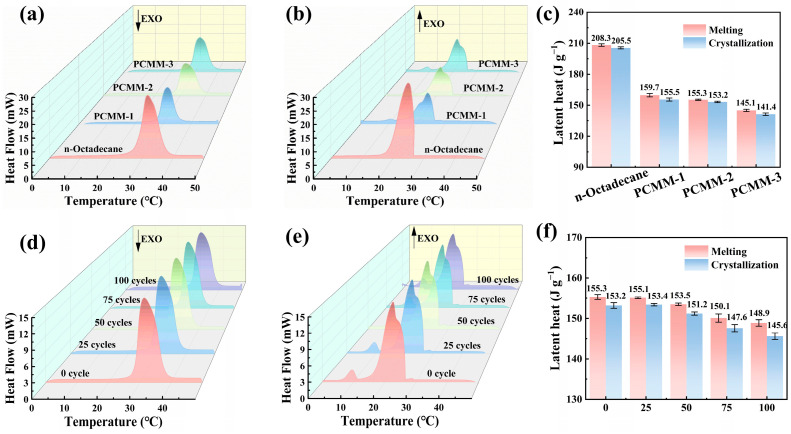
DSC (**a**) melting, (**b**) crystallization curves and (**c**) corresponding phase change enthalpies of n-octadecane and PCMM-1–PCMM-3. Thermal cycling stability of PCMM-2 showing the (**d**) melting curves, (**e**) crystallization curves, and (**f**) enthalpy variations over different thermal cycles.

The phase change enthalpies (Figure 4c) were obtained by integrating the DSC peaks, and the corresponding thermal properties are summarized in Table 1. The melting enthalpy (Δ$H_{m}$) and crystallization enthalpy (Δ$H_{c}$) of n-octadecane were 208.3 J g^−1^ and 205.5 J g^−1^, respectively. For the phase change microcapsules, the latent heat decreased as particle size decreased, mainly because smaller capsules usually contained a higher shell fraction and stronger interfacial confinement [38]. The supercooling behavior calculation are discussed in Supplementary Discussion S1, and the relevant figure is shown in Figure S1. To quantitatively assess the encapsulation performance of the microcapsules, the PCMs content ($C_{PCM}$ (%)) and the encapsulation efficiency (EE (%)) of PCMM were determined according to the following equations [39]:

(1) $$C_{PCM}=\frac{{\Delta H}_{m,PCMM}+{\Delta H}_{c,PCMM}}{{\Delta H}_{m,PCM}+{\Delta H}_{c,PCM}}\times 100\%$$

(2) $$EE=\frac{W_{PCMM}\times {\Delta H}_{PCMM}}{W_{PCM}\times {\Delta H}_{PCM}}\times 100=\frac{W_{PCMM}}{W_{PCM}}C_{PCM}(\%)$$

where ${\Delta H}_{m,PCMM}$ and ${\Delta H}_{m,PCM}$ are the melting enthalpies of the microcapsules and n-octadecane, respectively. ${\Delta H}_{c,PCMM}$ and ${\Delta H}_{c,PCM}$ are the enthalpies of crystallisation for microcapsules and n-octadecane, respectively. $W_{PCMM}$ is the measured mass of the purified and freeze-dried microcapsule product, and $W_{PCM}$ is the initial mass of n-octadecane used during preparation. The results are summarized in Table 1. As the particle size decreases, both $C_{PCM}$ and EE gradually decrease. This trend indicates that smaller microcapsules possess a higher shell-to-core mass ratio, leading to a lower effective PCMs fraction. In addition, the reduced EE may be related to increased material loss during centrifugation, washing, and freeze-drying when the microcapsule size becomes smaller. Therefore, under the condition of consistent chemical composition and crystal structure, the differences in latent heat among PCMM-1–PCMM-3 are mainly attributed to the variation in effective PCMs content and encapsulation efficiency.

Considering the subsequent need to balance latent heat and transparency, PCMM-2 was selected as the representative sample for thermal cycling stability evaluation. The DSC curves are shown in Figure 4d,e, and the corresponding thermodynamic data are summarized in Figure 4f. After 100 melting/crystallization cycles, the Δ$H_{m}$ of PCMM-2 decreased from 155.3 J g^−1^ to 148.9 J g^−1^, and the Δ$H_{c}$ decreased from 153.2 J g^−1^ to 145.6 J g^−1^, with corresponding latent heat retention rates of approximately 95.9% and 95.0%, respectively. The DSC peak position and shape only showed slight changes, indicating that the dense SiO_2_ shell can effectively stabilize the molten core material and reduce the risk of structural instability during repeated phase transitions. The leakage resistance of n-octadecane and the encapsulated microcapsules was investigated by a time-dependent heating test.

As shown in Figure S2, n-octadecane rapidly melted and spread on the substrate during heating, forming obvious wet stains after the solid–liquid phase transition. In contrast, PCMM-1, PCMM-2, and PCMM-3 maintained their powder-like morphology throughout the heating process from 0 to 210 s, with no visible liquid leakage or spreading observed. This result confirms that the SiO_2_ shell can effectively confine the molten n-octadecane core and act as a robust physical barrier against leakage, thereby endowing the microcapsules with excellent form stability during repeated phase transitions.

### 3.4. Optical Properties of Composites

The optical transmittance of the composites is mainly determined by the intrinsic transparency of the polymer matrix and the light scattering induced by the dispersed microcapsules, which are closely related to the microcapsule size, filler loading, and sample thickness. According to the formulations listed in Table S2, a series of acrylate matrices were prepared and compared in terms of optical transmittance. Among them, the cured poly(dodecyl acrylate) exhibited the highest transmittance at 550 nm (Figure S3) and was therefore selected as the matrix for the subsequent composite system.

**Figure 5:**
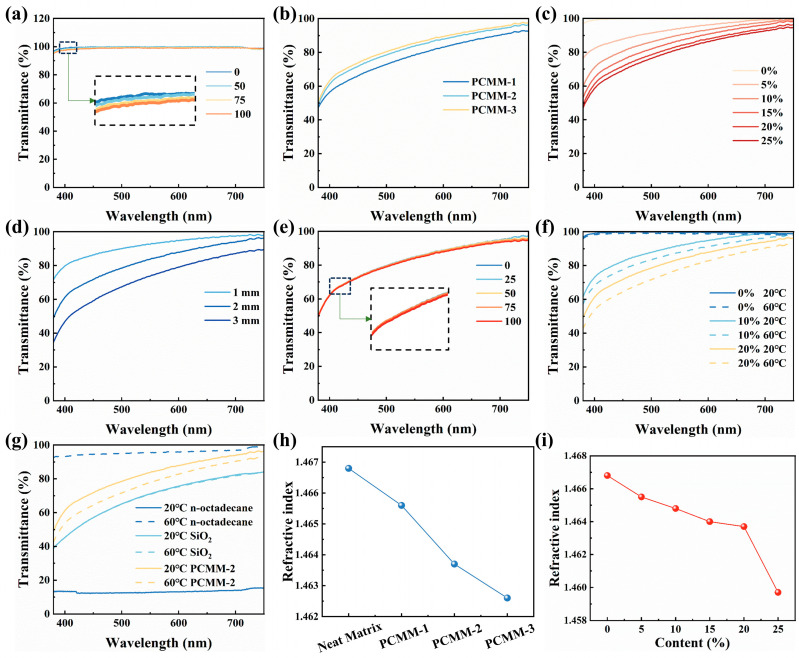
(**a**) Cyclic transmittance of neat matrix. Transmittance of samples incorporating fillers with (**b**) PCMM-1–PCMM-3, (**c**) different PCMM-2 loadings and (**d**) different thicknesses. (**e**) Cyclic transmittance of CPCMM. Transmittance of samples with (**f**) different PCMM-2 loadings (**g**) containing n-octadecane, SiO_2_, and PCMM-2 at 20 and 60 °C. (**h**) The refractive index of neat matrix and composites containing and PCMM-1–PCMM-3 and (**i**) its variation as a function of PCMM-2 loading.

The optical transmittance properties of the composites were characterized by UV–Vis spectroscopy. As shown in Figure 5a, only a 0.93% variation in transmittance was observed for the neat matrix after 100 heating–cooling cycles, indicating that it has good optical stability. At a fixed thickness of 2 mm and a filler content of 20 wt.%, the transmittance values at 550 nm for the composites containing PCMM-1, PCMM-2, and PCMM-3 were 78.42%, 83.75%, and 85.41%, respectively (Figure 5b). To further clarify the particle size dependence of optical transparency, transmittance at 550 nm was plotted as a function of the average microcapsule diameter in the inset of Figure 5b. Transmittance increased with a decreasing microcapsule diameter, suggesting that smaller microcapsules are more favorable for reducing the effective size of scattering centers and suppressing visible light scattering under the refractive-index-matched composite system. This size-dependent optical behavior is further supported by the refractive index results shown in Figure 5h. Considering the balance between optical transparency and latent heat storage, PCMM-2 was selected as the optimized filler. When the PCMM-2 loading was increased from 0 to 25 wt.%, the transmittance at 550 nm decreased from 99.73% to 81.85% (Figure 5c). Similarly, when the sample thickness was increased from 1 to 3 mm, transmittance decreased from 92.60% to 73.79% (Figure 5d). Therefore, 20 wt.% PCMM-2 and a thickness of 2 mm were chosen as the optimized condition, and the corresponding composite was denoted as CPCMM-2. The photograph of CPCMM-2 shown in Figure S4 confirms good visual transparency. After 100 heating–cooling cycles, only a 0.35% transmittance loss was observed for CPCMM-2 (Figure 5e).

To characterize the microstructural differences before and after compounding, the neat matrix and CPCMM-2 were observed by SEM. Figure S5a shows that the surface of the neat matrix is continuous, dense, and highly uniform, with only a few shallow, gentle stripes. Figure S5b shows the surface morphology of CPCMM-2, whose surface roughness significantly increases, exhibiting numerous irregular particle-like protrusions, aggregated structures, and wrinkled textures. Furthermore, when the microcapsule content is increased to 25 wt.%, obvious aggregation occurs, as shown in Figure S5c. The presence of these aggregated particles enhances light scattering, but the microcapsule particle size and refractive index are well matched with the substrate, avoiding severe scattering effects.

To evaluate the effect of the phase transition process on the optical transmittance of the composites, the transmittance of CPCMM-2 was measured at 20 °C (before phase transition) and 60 °C (after phase transition). As shown in Figure 5f, the transmittance of the pure base material hardly changed with temperature, while the samples with 10% and 20% microcapsules showed about a 5% decrease in transmittance after the phase transition. This is due to changes in refractive index and density of n-octadecane during the solid–liquid phase transition, which causes the effective refractive index matching between the core material and the SiO_2_ shell, and between the microcapsules and the acrylic ester matrix, to shift with temperature. This increases the refractive index difference at the interface and enhances scattering. It is worth noting that the decrease in transmittance is still relatively small, indicating that the dense SiO_2_ shell can effectively constrain the macroscopic morphology of the core material, suppress leakage and phase separation, and prevent the formation of stronger scattering centers during the phase transition. Therefore, the composite material maintains overall transparency before and after the phase transition, with only limited temperature responsiveness. Figure S6 visually verifies the retention of its clarity.

To verify the role of microcapsule encapsulation in maintaining transparency, three comparative systems with a thickness of 2 mm and a filler content of 20 wt.% were prepared using n-octadecane, SiO_2_ particles, and PCMM-2, respectively, and their transmittance values were measured at 20 °C and 60 °C for comparison. The transmittance spectra are shown in Figure 5g, with the corresponding photographs shown in Figure S7. The sample with directly added n-octadecane exhibited a significant decrease in transmittance after heating, reflecting phase separation and strong scattering caused by the melting and migration of the PCMs. In contrast, the SiO_2_-filled composite showed almost no change in transmittance with temperature, which can be attributed to the good refractive index matching between SiO_2_ and the polymer matrix and the absence of phase transition. Meanwhile, the PCMM-2-filled composite showed only an approximately 5% change in transmittance before and after the phase transition, while the background pattern remained clearly visible. This indicates that microcapsule encapsulation not only achieves the morphological stability of PCMs but also effectively suppresses scattering through size regulation and refractive index matching, thereby maintaining good transparency before and after the phase transition.

To further elucidate the transparency variation in the composites from the perspective of optical constants, the refractive indices of the samples were measured. As shown in Figure 5h, the refractive index of the neat matrix is the highest. After microcapsules were incorporated, the refractive index of the system decreased overall, following the trend matrix > PCMM-1 > PCMM-2 > PCMM-3. This indicates that the introduction of microcapsules reduces the effective refractive index of the composite system, and microcapsules with smaller particle sizes have a more significant effect on the refractive index. Figure 5i shows that, with the same microcapsule particle size, the refractive index gradually decreases as the microcapsule content increases. This is because the refractive index of the microcapsules is lower than that of the base material, and a higher microcapsule content causes more light scattering and energy loss, which leads to a gradual decrease in the refractive index of the composite material. In addition, filler aggregation may amplify local refractive index fluctuations and generate larger scattering centers.

### 3.5. Thermal and Mechanical Properties of Composites

**Figure 6:**
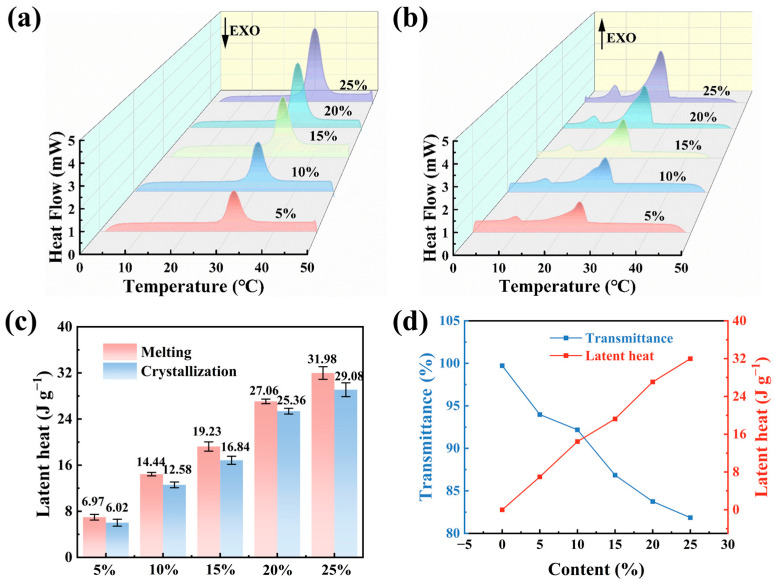
DSC (**a**) melting and (**b**) crystallization curves, (**c**) corresponding phase change enthalpies, and (**d**) correlation between optical transmittance and latent heat of CPCMM with different PCMM-2 loadings.

To characterize the effect of microcapsule loading on the thermal response of the transparent phase change composites and to establish its correlation with optical transmittance, the phase transition behavior of the composite systems was analyzed by DSC. As shown in Figure 6a,b, the melting and crystallization peak positions of composites with different PCMM-2 contents are essentially consistent, indicating that the neat matrix does not participate in the phase transition and serves only as a continuous supporting network. As the microcapsule content increased from 5 wt.% to 25 wt.%, the heat flow of the melting peak significantly increased, and the peak shape changed from broad and flat to sharp, reflecting an increase in core material proportion, enhanced intermolecular interactions, and a more concentrated phase transition process. Figure 6c shows the variations in Δ$H_{m}$ and Δ$H_{c}$ of the composites as a function of microcapsule loading. Both increase with the microcapsule content, but at 25 wt.%, the rate of enthalpy increase slows significantly compared to 20 wt.%, which is consistent with the decrease in effective core material proportion due to microcapsule agglomeration and slight shell damage at higher contents.

The effects of microcapsule loading on transmittance and enthalpy are shown in Figure 6d. With the increase in content, enthalpy increases while transmittance decreases, reflecting the trade-off between material transparency and thermal management. It can be observed that when the content reaches 20 wt.%, an optimal balance between enthalpy and transmittance is achieved. Further increases in content result in a larger decrease in transmittance, while the increase in enthalpy slows down, leading to a decrease in overall performance. The cyclic stability of the optimized CPCMM-2 sample was further evaluated, and the corresponding DSC curves and variations in phase change parameters and optical transmittance are shown in Figure S8 and Table S3. A detailed discussion is provided in Supplementary Discussion S2.

**Figure 7:**
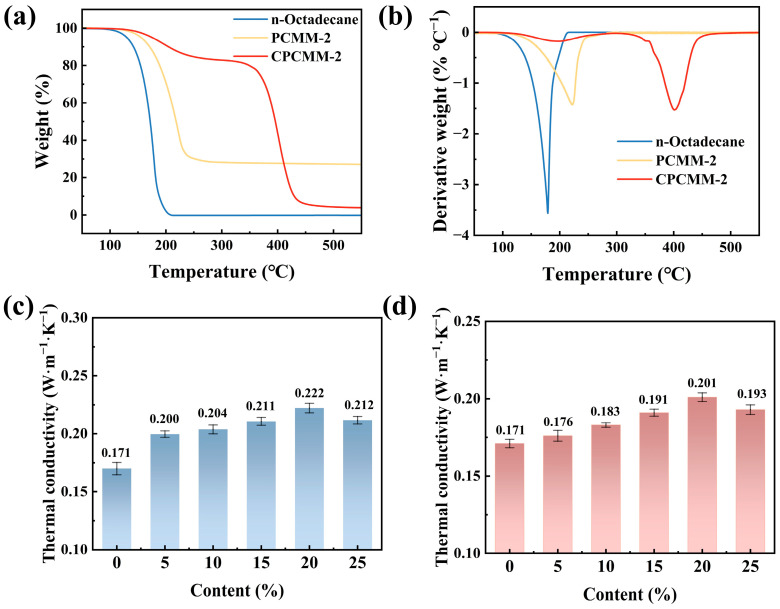
(**a**) TGA and (**b**) DTG curves of n-octadecane, PCMM-2, and CPCMM-2. Thermal conductivities of the composites as a function of PCMM-2 loading: (**c**) before phase transition and (**d**) after phase transition.

To investigate the influence of microcapsule incorporation on the thermal stability and heat-transfer behavior of the composites, thermogravimetric analysis (TGA), derivative thermogravimetry (DTG), and thermal conductivity measurements were performed. As shown in the TGA and DTG curves in Figure 7a,b, the main weight loss process of PCMM-2 and CPCMM-2 shifted to higher temperatures compared with n-octadecane, accompanied by a markedly reduced maximum weight loss rate. This result indicates that the encapsulation structure and composite matrix can effectively improve the thermal stability of n-octadecane. It should be noted that the shift in degradation temperature reflects delayed volatilization and thermal decomposition rather than an increase in the intrinsic melting temperature of n-octadecane. For PCMM-2, the SiO_2_ shell acts as a physical barrier that restricts the diffusion and volatilization of the molten PCMs, thereby enhancing the high-temperature morphological stability of the microcapsules. For CPCMM-2, the further improvement can be attributed to the additional confinement effect of the crosslinked polymer matrix, which provides secondary protection and further suppresses PCMs diffusion within the composite system.

As shown in Figure 7c,d, the thermal conductivity of the composites was measured under two temperature gradients, with the lower–upper surfaces maintained at 10–30 °C and 40–60 °C, respectively. Under the 10–30 °C condition, where n-octadecane mainly remained in the solid state, the thermal conductivity increased from 0.171 W m^−1^ K^−1^ for the pristine polymer matrix to 0.222 W m^−1^ K^−1^ at 20 wt.% PCMM-2. This improvement is attributed to the uniformly dispersed SiO_2_ shell, which provides additional solid-phase heat-transfer pathways and improves the effective thermal contact within the matrix. However, when the loading increased to 25 wt.%, the thermal conductivity decreased slightly to 0.212 W m^−1^ K^−1^, mainly due to microcapsule aggregation, local defects, and increased interfacial thermal resistance at excessive filler content. Under the 40–60 °C condition, where the encapsulated n-octadecane was in the liquid state, the composites showed a similar loading-dependent trend. The thermal conductivity increased from 0.171 to 0.201 W m^−1^ K^−1^ as the PCMM-2 content increased to 20 wt.% and then decreased to 0.193 W m^−1^ K^−1^ at 25 wt.%. The slightly lower thermal conductivity compared with that measured at 10–30 °C may be related to the lower effective heat-transfer ability of molten n-octadecane and the increased interfacial thermal resistance induced by the solid–liquid phase transition. The 20 wt.% PCMM-2 composite exhibited the optimal thermal conductivity under both temperature gradients, confirming its balanced filler dispersion and heat-transfer performance.

Tensile tests were conducted to assess the structural reliability of the composites for smart window and flexible thermal management applications (Figure S9 and Table S4). With PCMM-2 loading increasing from 0 to 20 wt.%, tensile stress, elongation at break, toughness, and modulus all increased, showing that well-dispersed microcapsules can reinforce the crosslinked acrylate network while maintaining deformability. The 20 wt.% composite showed the best overall mechanical performance, with a tensile stress of 0.247 MPa, elongation at break of 545.08%, toughness of 1.051 MJ m^−3^, and modulus of 2.64 kPa. At 25 wt.% loading, these values decreased markedly because microcapsule aggregation and interfacial defects weakened stress transfer. Additional discussion on mechanical limitations is provided in Supplementary Discussion S3.

### 3.6. Thermal Management Characteristics of CPCMM-2

**Figure 8:**
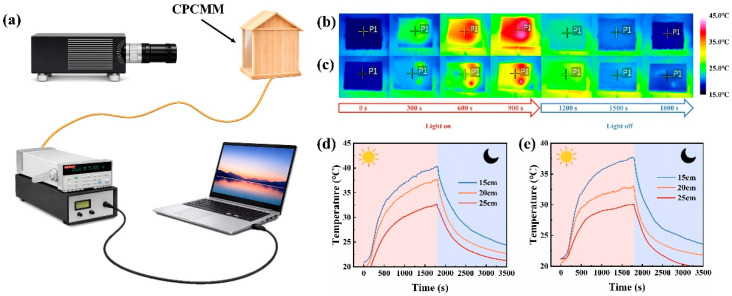
(**a**) Schematic illustration of the practical simulation for CPCMM-2. Infrared thermal images of (**b**) neat matrix and (**c**) CPCMM-2 under light irradiation. Real-time temperature profiles of (**d**) neat matrix and (**e**) CPCMM-2 during practical application.

To evaluate the practical thermal management capability of CPCMM-2, a xenon lamp was used as a simulated solar irradiation source to heat CPCMM-2 and neat matrix under identical conditions, while an infrared thermal imager continuously recorded the time-resolved evolution of the surface temperature. As shown in Figure 8b,c, after 900 s of continuous irradiation, the surface temperature of CPCMM-2 reached 35.8 °C, which was 5.4 °C lower than the temperature of 41.2 °C recorded for the neat matrix, indicating that CPCMM-2 effectively suppressed the temperature rise under photothermal loading. The light source was then switched off and the samples were allowed to cool naturally under ambient conditions for 900 s; the CPCMM-2 surface temperature remained at 19.8 °C, whereas that of the neat matrix decreased to 17.2 °C. These results indicate that, due to latent heat absorption/release during the phase transition, CPCMM-2 exhibited enhanced thermal inertia and temperature-buffering behavior during both heating and cooling, thereby reducing transient temperature fluctuations.

To further verify the application potential of CPCMM-2 in a practical scenario, a model house platform was constructed to examine indoor temperature responses (Figure 8a). The model house was built from wooden boards, and the inner surfaces were lined with thermal insulation cotton to approximate the insulation condition of building envelopes; CPCMM-2 was installed at an opening on one side of the house as a smart window component. In the test, a xenon lamp was used to simulate sunlight, and a Keithley temperature measurement system synchronously monitored the indoor temperature as a function of time to quantify the indoor thermal regulation performance under irradiation. The model house was positioned at distances of 15, 20, and 25 cm from the light source, irradiated for 1500 s, and then allowed to cool until 3500 s. The resulting temperature–time profiles are shown in Figure 8d,e, where Figure 8d corresponds to the control house equipped with the neat matrix window and Figure 8e corresponds to the house equipped with the CPCMM-2 smart window. The incorporation of the CPCMM-2 smart window markedly reduced the peak indoor temperature during heating. At 1800 s, the maximum temperatures in the control group were 40.21, 37.72, and 32.59 °C, whereas the corresponding values in the CPCMM-2 group decreased to 37.66, 33.00, and 30.11 °C. During the cooling stage, the indoor temperatures of the CPCMM group were higher than those of the control group, increasing from 23.56, 21.80, and 20.75 °C (neat matrix) to 24.40, 22.67, and 21.22 °C (CPCMM-2), respectively.

Collectively, these results demonstrate that CPCMM-2, while maintaining high optical transparency, suppressed indoor temperature rise and buffered thermal fluctuations via latent heat storage/release, thereby delivering effective passive thermal management under simulated solar irradiation. This provides experimental evidence supporting CPCMM-2 as a transparent temperature regulation material for smart window applications.

The optical performance of CPCMM-2 was also compared with representative PCM-based smart window systems, as summarized in Table S5. Reported systems, including PEG-based transparent wood [28], thermo-reversible transparent wood [40], CNT/paraffin–PDMS composites [41], and passive-dimming polymer/PCMs composites [42,43], have demonstrated promising passive optical modulation and thermal regulation functions. However, some of these materials still suffer from relatively low visible light transmittance or pronounced transmittance fluctuation during the phase transition process. In comparison, CPCMM-2 maintains a high visible light transmittance of 83.75% at 550 nm and shows only limited optical fluctuation before and after phase transition. More importantly, after 100 heating–cooling cycles, the optical attenuation of CPCMM-2 is only 0.35%, indicating excellent cycling transmittance stability. These results suggest that the refractive-index-matching strategy used in this work is effective for maintaining optical clarity and long-term transmittance stability in PCM-based smart window materials.

## 4. Conclusions

This study aims to address the issues of leakage and optical instability in organic PCMs within optical devices and to develop a smart window composite material with both high optical transparency and efficient passive thermal management. We propose and validate the hypothesis that by constructing submicron n-octadecane@SiO_2_ core–shell microcapsules and embedding them into a UV-curable poly(dodecyl acrylate) with a highly matched refractive index, we can synergistically suppress phase change-induced light scattering and core material leakage. The results demonstrate that this multi-scale optical synergy design endows the composite material with outstanding overall performance. At the optimal loading of 20 wt.%, the composite system not only achieves ideal phase change latent heat, enhanced thermal conductivity, and excellent mechanical toughness, but also maintains a visible light transmittance of up to 83.75% at 550 nm. The change in transmittance before and after phase change is only 5%, and the light transmittance degradation is just 0.35% after 100 thermal cycles, demonstrating exceptional optical and morphological stability. This study deepens the interface optical matching mechanism of transparent phase change composite systems, providing a highly promising strategy for the large-scale production of high-transparency, leakage-resistant energy-efficient building materials.

## Figures and Tables

**Table 1:** Properties of n-octadecane and PCMMs.

Sample	$T_{m}$ (°C)	$\Delta H_{m}$ (J g^−1^)	$T_{c}$ (°C)	$\Delta H_{c}$ (J g^−1^)	ΔT (°C)	$C_{PCM}$ (%)	EE (%)
n-Octadecane	32.73	208.3	23.29	205.5	9.4	-	-
PCMM-1	32.93	159.7	21.71	155.5	11.2	76.17	48.71
PCMM-2	33.04	155.3	21.42	153.2	11.6	74.55	46.73
PCMM-3	33.17	145.1	21.54	141.4	11.6	69.24	43.54

## Data Availability

The original contributions presented in the study are included in the article; further inquiries can be directed to the corresponding authors.

## Supplementary Materials

The following supporting information can be downloaded at https://www.mdpi.com/article/10.3390/nano16110648/s1, Figure S1: ΔT of n-octadecane and PCMM-1–PCMM-3; Figure S2 Time-dependent leakage-resistance test of n-octadecane and PCMM-1–PCMM-3 under continuous heating from 0 to 210 s; Figure S3: Transmission spectra of different polyacrylic acids; Figure S4. Photographs of the optimized CPCMM-2; Figure S5. SEM images of (a) neat matrix, (b) CPCMM-2, and (c) the composite containing 25 wt.% PCMM-2; Figure S6. Photographs showing the optical transparency of composites with different PCMM-2 loadings at (a) 20 °C and (b) 60 °C; Figure S7. Photographs showing the optical transparency of composites with 20 wt.% filler loading: SiO2-filled composite at (a) 20 °C and (c) 60 °C, n-octadecane-filled composite at (b) 20 °C and (d) 60 °C; Figure S8. Thermal cycling stability of the optimized CPCMM-2 composite: DSC (a) melting and (b) crystallization curves recorded after 0, 25, 50, 75, and 100 heating–cooling cycles, and (c) corresponding melting and crystallization enthalpies; Figure S9. Tensile properties of CPCMMs with different PCMM-2 loadings: (a) stress–strain curves; (b) tensile stress and toughness; (c) elongation at break and modulus; Table S1. Reaction parameters for PCMMs; Table S2. Relevant parameters for composite materials; Table S3. The cyclic performance of CPCMM-2; Table S4. Mechanical properties of PCMM-2 at different concentrations; Table S5. Comparison of the optical and thermal performances of CPCMM-2 with recently reported PCM-based smart-window systems; References [44,45,46,47,48,49] are cited in the Supplementary Materials.

## Abbreviations

The following abbreviations are used in this manuscript:

PCMs	Phase change materials
TEOS	Tetraethyl orthosilicate
CTAB	Hexadecyl trimethyl ammonium bromide
VTES	Vinyltriethoxysilane
DEAP	2,2-Diethoxyacetophenone
EGDMA	Ethylene glycol dimethacrylate
SEM	Scanning electron microscopy
XRD	X-ray powder diffractometer
FT-IR	Fourier transform infrared spectroscopy
DSC	Differential scanning calorimetry
UV–Vis	Ultraviolet–visible spectroscopy
TGA	Thermogravimetric analysis
DTG	Derivative thermogravimetry
O/W	Oil/water
