# Supplementary material for: Highly Transparent Phase Change Smart Windows Enabled by Refractive-Index-Matched n-Octadecane@SiO2 Microcapsule Composites
Source: Nanomaterials (Basel). 2026 May 22;16(11):648. doi: 10.3390/nano16110648 (PMC13257580; doi:10.3390/nano16110648)
Supplement: Supplementary file 1 [file nanomaterials-16-00648-s001.zip › nanomaterials-4313366-supplementary.pdf]

# Highly Transparent Phase Change Smart Windows Enabled by Refractive-Index-Matched n-Octadecane@$SiO_2$ Microcapsule Composites

*Fusen Yang[1], Zhixing Zhang[1], Yiyu Feng[1], Mengmeng Qin[1,]*, Wei Feng[1,2,]**

Mr. F. Yang, Dr. Z. Zhang, Prof. Y. Feng, Prof. M. Qin, Prof. W. Feng

[1] Tianjin Key Laboratory of Composite and Functional Materials, School of Materials Science and Engineering, Tianjin University, Tianjin 300350, China

Prof. W. Feng

[2]State Key Laboratory of Precious Metal Functional Materials, Tianjin University, Tianjin 300350, China

*E-mail: qmm@tju.edu.cn (Mengmeng Qin)

weifeng@tju.edu.cn (Wei Feng)

**Table S1.** Reaction parameters for PCMMs.

| Samples | Water: ethanol | Water (mL) | Ethanol (mL) | n-Octadecane (g) | TEOS (mL) | CTAB (g) | VTES (mL) | $NH_3{\cdot}H_2O$ (mL) |
|---|---|---|---|---|---|---|---|---|
| PCMM-1 | 1.5:1 | 25.6 | 17.0 | 3 | 1.8 | 0.328 | 0.2 | 0.52 |
| PCMM-2 | 2:1 | 28.4 | 14.2 | 3 | 1.8 | 0.328 | 0.2 | 0.52 |
| PCMM-3 | 2.5:1 | 30.4 | 12.2 | 3 | 1.8 | 0.328 | 0.2 | 0.52 |

## S1. Supercooling discussion

The degree of supercooling (ΔT = Tm − Tc) of pristine n-octadecane and the encapsulated microcapsules is shown in Figure S1. The ΔT of n-octadecane is 9.4 °C. After encapsulation, the degree of supercooling of the microcapsule samples increased. This increase in ΔT can be attributed to the confinement and interfacial modulation effects introduced by the $SiO_2$ shell, which alter the nucleation pathway of n-octadecane and reduce the probability of spontaneous nucleation, thus requiring a higher thermodynamic driving force to trigger crystallization [44]. For smart-window applications, increased supercooling may delay crystallization and latent-heat release during cooling. Consequently, the heat-release process may occur at a slightly lower temperature, which could weaken the immediate thermal-buffering response when the indoor temperature begins to decrease. Nevertheless, the crystallization temperature of PCMM-2 remains around 21.4 °C, which is close to the indoor thermal-comfort range. Therefore, although the increased supercooling may introduce a delayed heat-release behavior, the CPCMM system can still provide useful temperature regulation by slowing down temperature fluctuations during the cooling stage.

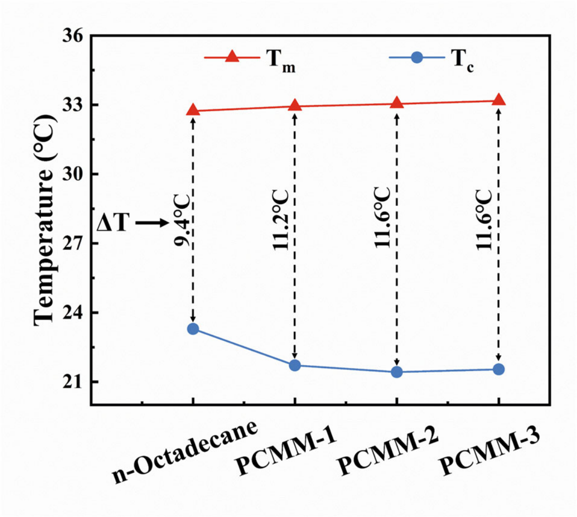


**Figure S1.** ΔT of n-octadecane and PCMM-1–PCMM-3.

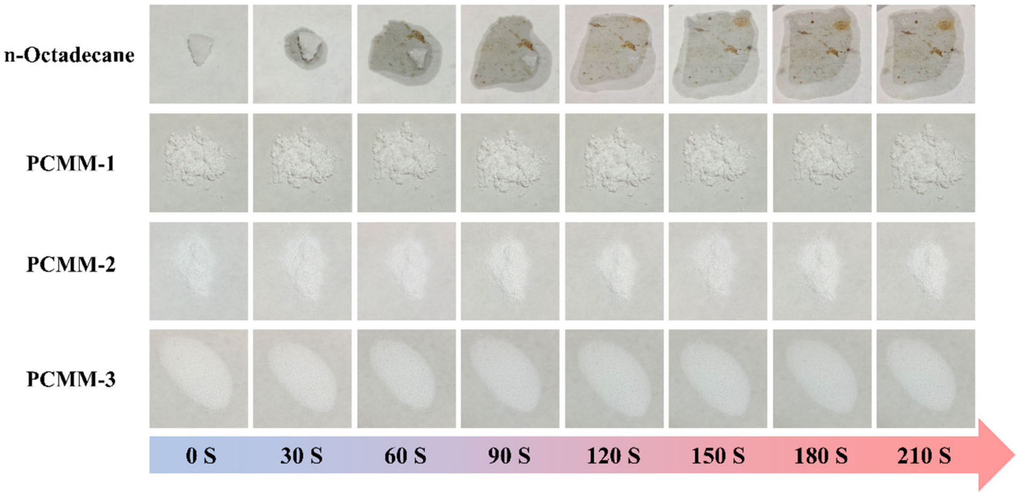


**Figure S2.** Time-dependent leakage-resistance test of n-octadecane and PCMM-1–PCMM-3 under continuous heating from 0 to 210 s.

**Table S2.** Relevant parameters for composite materials.

| | dodecyl acrylate (g) | n-octyl acrylate (g) | n-butyl acrylate (g) | EGDMA (μL) | DEAP (μL) |
|---|---|---|---|---|---|
| poly(dodecyl acrylate) | 4 | - | - | 20 | 10 |
| poly(n-octyl acrylate) | - | 4 | | 20 | 10 |
| poly(n-butyl acrylate) | - | - | 4 | 20 | 10 |

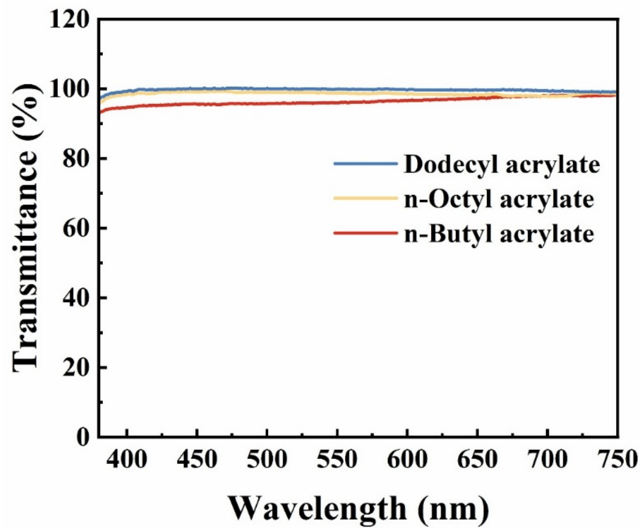


**Figure S3.** Transmission spectra of different polyacrylic acids.

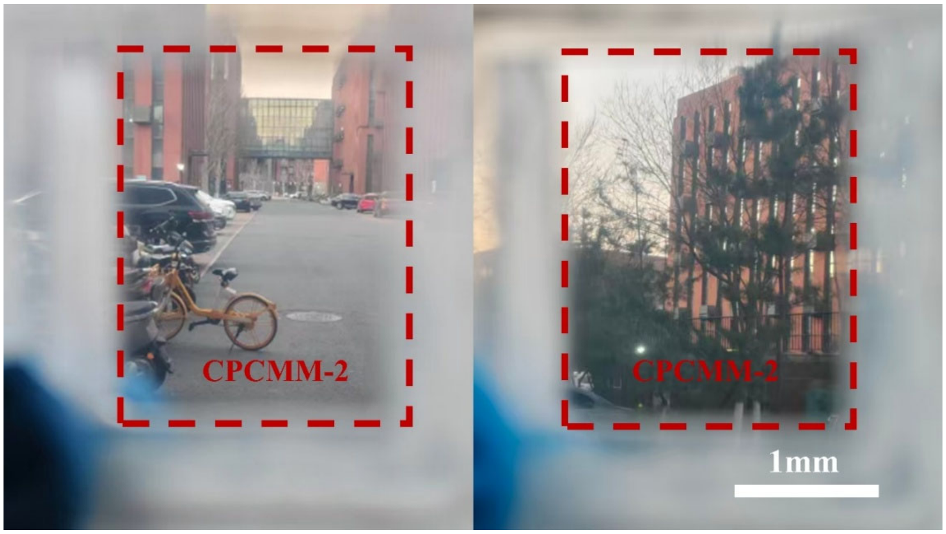


**Figure S4.** Photographs of the optimized CPCMM-2.

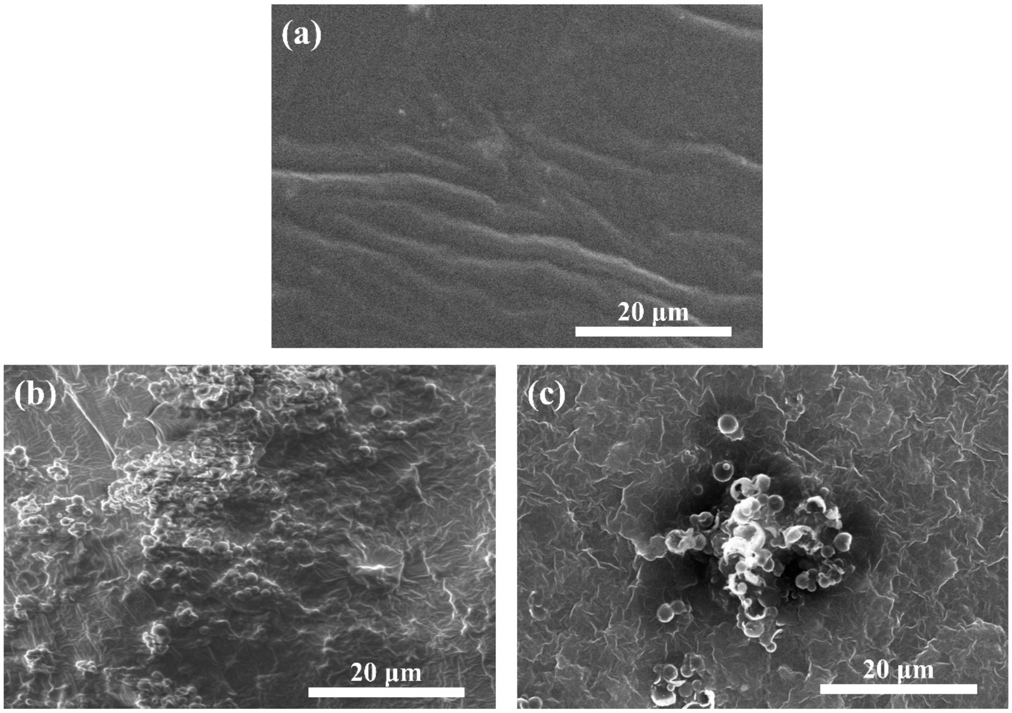


**Figure S5.** SEM images of (a) neat matrix, (b) CPCMM-2, and (c) the composite containing 25 wt.% PCMM-2.

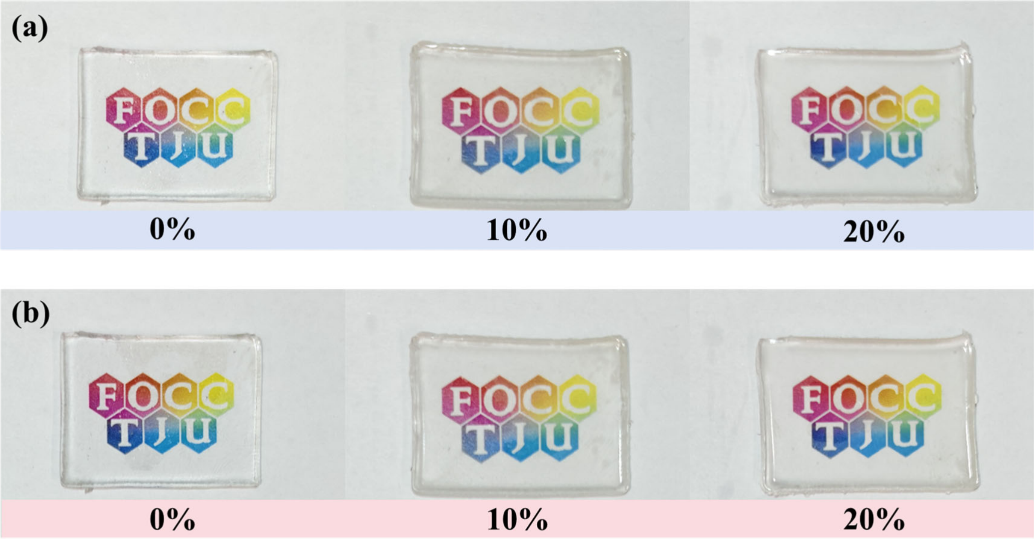


**Figure S6.** Photographs showing the optical transparency of composites with different PCMM-2 loadings at (a) 20 °C and (b) 60 °C.

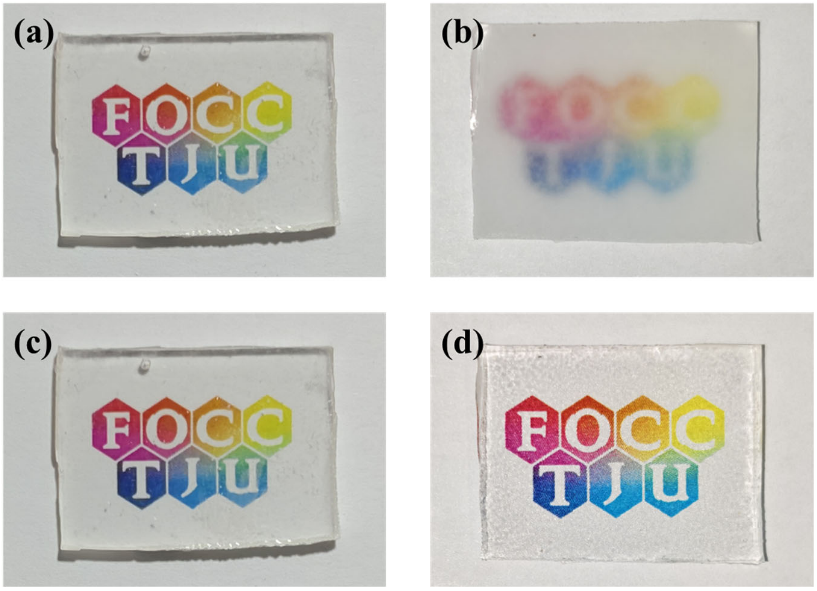


**Figure S7.** Photographs showing the optical transparency of composites with 20 wt.% filler loading: $SiO_2$-filled composite at (a) 20 °C and (c) 60 °C, n-octadecane-filled composite at (b) 20 °C and (d) 60 °C.

**S2. Cyclic stability of CPCMM-2**

The cyclic stability of the optimized CPCMM-2 sample was evaluated by monitoring its DSC curves, phase-change parameters, and optical transmittance during repeated heating–cooling cycles. As shown in Figure S8, the melting and crystallization curves of CPCMM-2 maintained similar peak positions and overall shapes after cycling, indicating that the phase-transition behavior remained stable during repeated thermal treatment. The corresponding thermal parameters are summarized in Table S3. After 100 cycles, the melting temperature changed slightly from 33.04 °C to 32.98 °C, while the crystallization temperature changed from 21.42 °C to 21.32 °C. Meanwhile, the melting enthalpy decreased only slightly from 27.06 J $g^{-1}$ to 26.83 J $g^{-1}$, and the crystallization enthalpy decreased from 25.36 J $g^{-1}$ to 25.13 J $g^{-1}$, corresponding to enthalpy retention values above 99%.

In addition to the stable phase-change behavior, CPCMM-2 also exhibited excellent cycling optical stability. The transmittance decreased only slightly from 84.75% to 84.51% after 100 heating–cooling cycles, indicating that repeated phase transition did not cause obvious leakage, phase separation, or severe microstructural deterioration. This stable thermal–optical cycling performance can be attributed to the confinement effect of the $SiO_2$ shell and the refractive-index matching between the microcapsules and the acrylate matrix. Therefore, the optimized CPCMM-2 composite can maintain both phase-change performance and optical transparency after repeated thermal cycling, supporting its potential use as a transparent phase-change functional layer for smart-window applications.

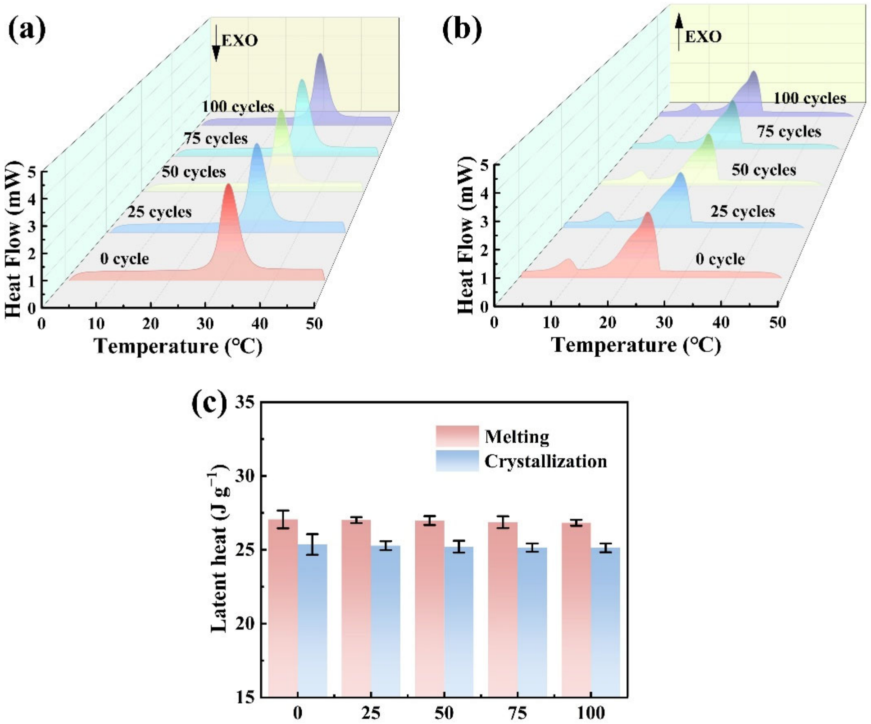


**Figure S8.** Thermal cycling stability of the optimized CPCMM-2 composite: DSC (a) melting and (b) crystallization curves recorded after 0, 25, 50, 75, and 100 heating–cooling cycles, and (c) corresponding melting and crystallization enthalpies.

**Table S3.** The cyclic performance of CPCMM-2.

| number of cycles | $T_m$ (℃) | $\Delta H_m$ (J g$^{-1}$) | $T_c$ (℃) | $\Delta H_c$ (J g$^{-1}$) | Transmittance (%) |
|---|---|---|---|---|---|
| 0 | 33.04 | 27.06 | 21.42 | 25.36 | 84.75 |
| 25 | 33.12 | 27.01 | 21.43 | 25.28 | 84.89 |
| 50 | 33.08 | 26.98 | 21.39 | 25.21 | 84.67 |
| 75 | 33.01 | 26.87 | 21.37 | 25.15 | 84.55 |
| 100 | 32.98 | 26.83 | 21.32 | 25.13 | 84.51 |

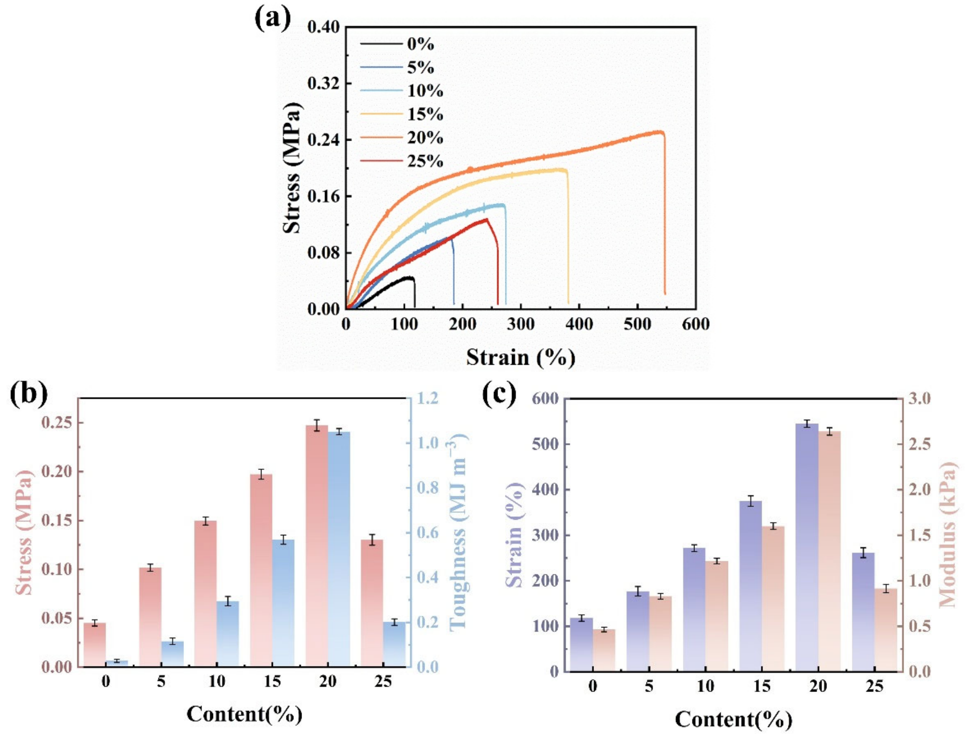


**Figure S9.** Tensile properties of CPCMMs with different PCMM-2 loadings: (a) stress–strain curves; (b) tensile stress and toughness; (c) elongation at break and modulus.

**Table S4.** Mechanical properties of PCMM-2 at different concentrations.

| Content (wt.%) | Stress (MPa) | Strain (%) | Toughness (MJ $m^{-3}$) | Modulus (kPa) |
|---|---|---|---|---|
| 0 | 0.04520 | 118.14 | 0.02885 | 0.46533 |
| 5 | 0.10171 | 176.91 | 0.11566 | 0.83090 |
| 10 | 0.14960 | 271.74 | 0.29492 | 1.21667 |
| 15 | 0.19727 | 375.07 | 0.56910 | 1.59951 |
| 20 | 0.24729 | 545.08 | 1.05113 | 2.64000 |
| 25 | 0.13016 | 261.58 | 0.20167 | 0.91413 |

**S3. Mechanical-property interpretation and application limitation**

The loading-dependent mechanical behavior of CPCMM composites can be mainly related to the dispersion state of PCMM-2 and its interaction with the crosslinked acrylate network. At moderate loading, the dispersed microcapsules can serve as reinforcing domains and contribute to stress transfer during deformation. Similar reinforcement effects have also been reported in polymer composites containing silica-based fillers, where filler dispersion, interfacial adhesion, and particle size play important roles in determining tensile properties [45, 46]. However, excessive microcapsule loading can lead to aggregation and interfacial defects, which act as stress-concentration sites and weaken effective load transfer. This explains the deterioration in mechanical performance observed at 25 wt.% PCMM-2 loading.

It should be noted that the tensile stress and modulus of CPCMM-2 are lower than those of conventional window interlayers such as poly(vinyl butyral) (PVB) [47], ethylene–vinyl acetate (EVA) [48], and ionoplast materials [49]. However, these materials have different application roles. Commercial interlayers are generally used as load-bearing safety components in laminated glass, where strong adhesion, impact resistance, and post-breakage integrity are required. In contrast, CPCMM-2 is designed as a transparent phase-change functional layer for passive thermal regulation rather than as a structural safety interlayer. Therefore, its mechanical properties should mainly ensure processability, flexibility, and dimensional stability during handling and potential integration with transparent substrates. Further mechanical reinforcement will still be necessary for large-area window applications, and future work may focus on strengthening the polymer network or improving interfacial bonding while maintaining optical transparency and latent-heat storage capability.

**Table S5.** Comparison of the optical and thermal performances of CPCMM-2 with recently reported PCM-based smart-window systems.

| System | transmittance before phase change | transmittance after phase change | Transmittance fluctuation across phase change | Latent heat (J $g^{-1}$) | Reference |
|---|---|---|---|---|---|
| This work | 83.75% | 78.92% | 4.83% | 27.06 | This work |
| PEG-based transparent wood | 62.0% | 68.0% | 6.0% | 76.00 | [28] |
| SBO5/TW | 23.7% | 74.9% | 51.2% | 7.20 | [40] |
| CNT/paraffin–PDMS | 0.7% | 67.0% | 66.3% | No mention | [41] |
| PBB/TD/CCD-2 | 0.20% | 74.5% | 74.3% | 161.9 | [42] |
| PBMA/MA-3 | 0.1% | 89.0% | 88.9% | 113.2 | [43] |

## References


44. Kadi K. E.; Murad S.; Janajreh I. Ice crystallization kinetics in supercooled droplets from a molecular perspective, J. Colloid Interface Sci. 2026, 703, 139192. https://doi.org/10.1016/j.jcis.2025.139192.
45. Moghaddam F.; Tutunchi A. The effect of modified silica nanoparticles on the mechanical properties of UV-curable polyurethane acrylate adhesive, Int. J. Adhes. Adhes. 2025, 137, 103899. https://doi.org/10.1016/j.ijadhadh.2024.103899.
46. Kontou E.; Christopoulos A.; Koralli P.; Mouzakis D. E. The Effect of Silica Particle Size on the Mechanical Enhancement of Polymer Nanocomposites, Nanomaterials 2023, 13, 1095. https://doi.org/10.3390/nano13061095.
47. Knight J. T.; El-Sisi A. A.; Elbelbisi A. H.; Newberry M.; Salim H. A. Mechanical behavior of laminated glass polymer interlayer subjected to environmental effects, Polymers 2022, 14, 5113. https://doi.org/10.3390/polym14235113.
48. Sable L., Kinsella D.; Kozłowski M. Influence of EVA, PVB and Ionoplast interlayers on the structural behaviour and fracture pattern of laminated glass, Int. J. Struct. Glass Adv. Mater. Res. 2019, 3, 62–78. https://doi.org/10.3844/sgamrsp.2019.62.78.
49. Offereins D.; Pauli A.; Siebert G. Mechanical performance of liquid cold-poured interlayer adhesives in comparison to PVB, EVA, and ionomers, Glass Struct. Eng. 2024, 9, 569–586. https://doi.org/10.1007/s40940-024-00274-z.
